# Tuberculosis1Abstract of address before “The Ploughman,” Farmers’ Meeting, Boston, April 6, 1895.

**Published:** 1895-06

**Authors:** Austin Peters


THE JOURNAL

OF

COMPARATIVE MEDICINE AND

VETERINARY ARCHIVES.

VOL. XVI. *JUNE*, 1895. No. 6.

### TUBERCULOSIS.[1]

BY AUSTIN PETERS, V.S.

It is several years since it has been my pleasure to address a *Ploughman* audience upon the subject of tuberculosis, but since then much that I told you at that time has turned out to be true, and new knowledge has been acquired, until this subject has attained so much importance as to be receiving a vast amount of public attention in our eastern dairy States, not only in New England, but in New York State and Pennsylvania as well, and, although present views and present methods of dealing with this malady may become modified in many ways as knowledge concerning it increases, yet it has secured a recognition that will never be lost sight of, and State governments having once taken steps for its suppression can never again ignore its existence and magnitude.

I shall probably this morning touch upon points which we have considered upon a former occasion, but a repetition of some features of our topic will not be time wasted, and in addition there are new facts to be studied which have developed the last two or three years.

It is sufficient to say that tubercle means a nodule, and that tuberculosis is a disease characterized by the formation of nodular growths in different tissues or organs of the animal economy, these nodules being small and of one consistency at first, later

[1] Abstract of address before "The Ploughman," Farmers' Meeting, Boston, April 6, 1895.

their centres break down, becoming first cheesy, and when older infiltrated with deposits of lime-salts, cutting then with a gritty feeling, as though they contained particles of sand. Under the microscope a tubercle is found to consist of cells, chiefly round cells, resembling white blood-corpuscles, among them a few large cells, known as "giant cells;" the external cells may be flattened by pressure, representing epitheliod cells. Scattered among the cells and sometimes within them, especially within the "giant cells," are found bacilli about 1-10,000 of an inch long, when properly stained having a characteristic staining reaction; looking, then, under a suitable microscope, like scratches made with a very fine crow-quill pen dipped in red ink, drawn upon a blue field. The adjective *tuberculous* is applied to animals suffering with tuberculosis, and *tubercular* to lesions resembling tuberculosis, but in reality nodular growths of a different origin. These two adjectives are, however, sadly mixed up by persons who ought to know the difference in their meaning. Until within the past few years different forms of tuberculosis were described by different names, and some of them were supposed to be even distinct diseases; thus consumption of the lungs is known as *phthisis;* consumption of the bowels as *tabes mesenterica;* tuberculosis of the lymphatic glands as *scrofula;* tuberculosis of the skin as *lupus;* then we have tuberculous *arthritis*, or tuberculosis of the joints; and tuberculous *mammatisi*, or tuberculous inflammation of the udder. *Lupus* and *scrofula*, for example, were formerly supposed to be distinct diseases, but are now recognized as tuberculous because it is found that they are caused by the bacillus of tuberculosis exactly as *phthisis* and *tabes* are.

*Etiology*. The etiology of a disease is the study of its cause; the cause of tuberculosis is the tubercle bacillus, as discovered by Koch; it has a certain appearance, a characteristic color-reaction, and can be artificially cultivated. It is identically the same germ in man and in the lower animals, as can be proved by the microscope, artificial cultivation, and inoculation experiments. The causes of a disease may be divided into the exciting cause and predisposing causes; these hold good in regard to any infectious malady. The exciting cause of tuberculosis is the specific germ which gives rise to it, the *bacillus tuberculosis*.

The following are the predisposing causes of bovine tuberculosis, being a modification of Walley's list:

First, species of animal; second, hereditary predisposition and congenital tuberculosis; third, breeding in and in; fourth, breed; fifth, early-, late-, and over-breeding; sixth, physical conformation; seventh, climate and locality; eighth, debility produced by lactation, sickness, and bad food; ninth, bad hygienic conditions.

Although statistics are meagre regarding the extent to which tuberculosis prevails among our herds, it is certain that it is far too prevalent. Among the cattle running upon the ranges of the great West it is practically unknown, but among the cows in the dairy sections of the East it is very common. During the winter of 1892–'93 I had the pleasure of acting as Chief Inspector of Cattle for the New York State Board of Health. Creatures were examined and condemned by physical examination only; the tuberculin test seemed to be still in an experimental stage, and it was thought best not to apply it just then. Two dairy sections were taken, one in Westchester county, the other in Orange county, for a general examination of the cows, about 10,000 being examined in each locality, with the result that nearly one per cent. were found to be tuberculous in Westchester county, and about one-third of one per cent. in Orange county. Taking for granted that the tuberculin test detects three or four cases to every one that can be ascertained by a physical examination, we find that about one per cent. of the cows in Orange county and three to four per cent in Westchester county must have been tuberculous. Orange county reminds one of Worcester county in this State, and the cows in Westchester county are under similar conditions to the cows in the suburbs of our eastern Massachusetts cities. Applying, then, these figures to our own State, it is a conservative estimate to put the per cent. of disease in Worcester county at one, and in the towns surrounding Boston at three or four per cent. If the Cattle Commissioners carry out the tuberculin test of the State which they have undertaken, these figures will be found too low rather than too high. Figures of three or four per cent. for our more thickly populated cattle districts coincide closely with the abattoir statistics of cattle slaughtered in the more populous European centres.

Tuberculosis is ordinarily acquired in one of three ways: 1, by inhalation; 2, by ingestion; 3, by inoculation.

Tuberculosis acquired by inhalation is the form seen in the great majority of cases, both in man and cattle, the tubercle bacilli gaining entrance to the lungs in the inspired air. Under these circumstances the lungs are probably affected primarily, and the disease spreads from there to other organs. Here the first symptom is the cough, and a man or other animal suffering from phthisis is constantly coughing up and expectorating (at least the man expectorates, animals simply cough out) morbid products from the lungs; this sputum contains numbers of tubercle bacilli, and when it dries and becomes part of the dust floating in the atmosphere the bacilli also float with the dust and may gain access to the lungs of healthy animals. Now, if the person or animal is just recovering from a severe cold and has a little ulcerated spot in the lung, the bacilli may gain a foothold there and grow and produce the disease, spreading from the original point to other parts of the lungs and to other organs. Or, if large numbers are present in the air of a room, a person constantly inhaling them may become infected, even though naturally quite healthy, being finally overcome by numbers. The bacilli of tubercle retain their vitality for a long time: Koch has found that after several months in a dry room they still were capable of producing the disease. Dr. A. K. Stone,[1] of Boston, Mass., has demonstrated that dried tubercle bacilli retain some of their virulence for at least three years.

Nearly every one has doubtless inhaled these bacilli, but when present in small numbers, and with the pulmonary mucous membrane healthy, the ciliated epithelium of the air-passages returns them to the throat, and they are expectorated. Then the colony is so slow in growing at first that it may have a difficult time in fixing itself permanently.

Phthisical patients in expectorating are constantly giving out germs which may infect other people, and possibly cattle; and *vice versa*, there is a possibility that a person of a cachetic diathesis working in a stable occupied by tuberculous cows may acquire phthisis from them.

Infection in this manner is the way in which tuberculosis is ordinarily acquired among the human family, and, from a public-health point of view, waging war upon tuberculous cows without taking measures for the isolation of tuberculous persons, the disinfection of premises where a person has just died of tuberculosis, or where a consumptive family has just moved out of a tenement, is simply the same thing as trying to stop a small leak at the spigot while allowing an enormous leak at the bung to go on unheeded.

[1] American Journal of the Medical Sciences, March, 1891.

Infection by ingestion means acquiring the disease through the digestive tract by swallowing the virus, and this method of infection is of special interest in a consideration of the malady from a veterinary sanitary standpoint, as it is from the use of the flesh and milk of tuberculous animals that the chief danger lies of infection by this method. Tuberculosis by ingestion may also result from secondary infection, a tuberculous patient swallowing his own sputum. Tuberculosis by ingestion may also be caused in pet dogs with morbid appetites, or in fowls, by swallowing the sputum of a consumptive person.

The work conducted under the auspices of the old Massachusetts Society for Promoting Agriculture, by Dr. Ernst and myself, showed that the milk from twenty-eight per cent. of the cows investigated contained the bacilli of tuberculosis, and that the germs were virile as shown by the results of the feeding and inoculation experiments; also that it was not necessary for a cow to have a diseased udder in order to be a source of danger. These results are borne out by the researches of various scientists, such as Bang, Bollinger, Pearson, and others.

A number of interesting cases have come to my personal knowledge of the infection and death of infants as a result of being fed upon the milk of a tuberculous cow, therefore the danger exists and should be guarded against.

On the other hand, it must be borne in mind that the cows experimented upon at Mattapan were all picked out by a physical examination as diseased (this was before the discovery of tuberculin), that tuberculosis in the cow is apt to assume a chronic form, and that many cows picked out by the tuberculin test appear on a physical examination healthy, and upon post-mortem have only very slightly diseased mediastinal glands (lymphatic glands between the lungs). I believe that animals as slightly diseased as that are of no immediate danger to other cattle or to the public health.

Although there may be danger from the meat of a badly diseased animal, there is probably very little from one slightly diseased, especially as meat is usually eaten cooked, and 170° Fahr. is sufficient to kill the tubercle bacillus. The great danger from a sanitary standpoint is from the uncooked milk of a badly diseased cow or one having a tuberculous udder. There is now a theory that the cooked meat or milk from a tuberculous creature contains tuberculin, and that therefore these products are dangerous to use, especially for consumptive persons in whom a dangerous reaction may be produced by the tuberculin present. This seems to me to be a far-fetched unproved hypothesis.

There is no reason that I can see why the meat of an animal but slightly tuberculous should not be used for beef, or why a very slightly diseased cow or ox may not be fattened for beef, under a proper system of abattoir inspection; and a law requiring the compulsory rendering or destruction of all such carcasses is unwise, injudicious, and a part of a very bad and wasteful system of political economy.

Besides the sanitary aspect of tuberculosis, there is also the farmer's point of view that it is a communicable disease from one cow to another, and the man owning a healthy herd wishes protection from buying from a neighbor a diseased cow which might contaminate his premises.

Infection may occur also from inoculation when the virus enters the body through the skin, and may be either accidental or intentional. It is intentional when tried experimentally upon animals, and accidental if the virus enters the system through a scratch or cut in the skin. Accidental inoculation might occur to a person who cut or scratched his hand while making an autopsy upon a tuberculous animal or while performing an operation upon a tuberculous subject. When the disease is induced by inoculation it begins with a nodule at the point of entrance, but if it is the result of ingestion the bacilli may enter the circulation and first attack some organ that has a lesser resisting power than other organs.

*Diagnosis.* The diagnosis of bovine tuberculosis is just now kicking up a good deal of a rumpus on account of the method used by our Cattle Commission; that is, by means of the tuberculin test; hence the tuberculin test is at the present time creating a great deal of interest among farmers and milkmen, and all sorts of questions are asked concerning its efficacy. Is it reliable? Does it do any injury to the animal? and similar queries arise by the score. Therefore, in closing this talk, the most profitable use to make of the remainder of the time at our disposal is, perhaps, to try and clear up some of these points. Until within a very few years diagnoses of tuberculosis in bovines were made by means of a physical examination, although in a few instances the microscope could be used to assist; but the physical signs are uncertain, and not infrequently other diseases may be confounded with the malady we are considering; now, however, we have a very valuable aid in what we know as the tuberculin test; I say aid, because I do not consider tuberculin infallible as a diagnostic agent, although the per cent. of errors made is very small when it is used under suitable conditions by a competent person.

Tuberculin is a chemical product of the tubercle bacillus. It is prepared by taking cultures of the germ which have grown long enough to produce their ptomaines, extracting these products by means of glycerin, filter the fluid and sterilize it; it contains no living germs and cannot by any possibility produce tuberculosis in a healthy animal.

It was first prepared by Koch, and was first introduced to the scientific world in 1890 as a cure for consumption in humans; it was not satisfactory, however, as a cure, and in many cases hastened death; it soon fell into disuse for the purpose for which it was originally intended; but it was found that consumptive patients after treatment had a characteristic febrile reaction; it was deduced from this that it would have a similar effect on cattle, and the use to which it is now put is that of a diagnostic agent for bovine tuberculosis, although I believe it has in some instances a curative effect upon this malady, and may possibly come into use again for the treatment of consumption.

As a diagnostic agent in cattle practice it is used in the following way: In the evening the animal's temperature is taken, then (for an averaged-sized cow) two and one-half cubic centimetres of a 10 per cent. solution of tuberculin, in sterilized water with 0.5 per cent. of carbolic acid in it, are injected under the skin over the anterior part of the shoulder by means of a good, strong hypodermic syringe. The temperature is again taken early the next morning and at intervals of two or three hours during the next day. It should have no effect on a healthy cow, but on a tuberculous one it ought to produce a marked febrile reaction between eight to twenty hours after the injection. To be very scientific, a number of temperatures can be taken during the day preceding the test to establish a mean normal temperature. If the animal has long hair it should be clipped off at the point of inoculation; and if the skin be dirty there it should be wiped clean. The animals should not receive any water during the test, as cold water lowers a rising temperature and disarranges matters; if the cows are given warm water daily it does no harm to water during the test, but they must not leave their stalls. I allow a little hay to keep them quiet, but some veterinarians allow no food until the test is over.

For illustration the following tables from my note-book will show the temperature of a healthy cow and one that reacts:

Healthy cow:

9 P.M., Temperature, 101° Fahr.

Tested with 2¾ c. c. of a 10 per cent. tuberculin solution:

| | Temperature. |
|---|---|
| 6.00 A.M. | 101.6° |
| 8.30 " | 101.8 |
| 11.00 " | 101.6 |
| 2.30 P.M. | 102.2 |
| 4.00 " | 101.8 |

Tuberculous cow:

Tested with 2¼ c. c. of a 10 per cent tuberculin solution:

| | Temperature. |
|---|---|
| 6.00 A M. | 101 8° |
| 8.30 " | 104.4 |
| 11.00 " | 106.8 |
| 2.30 P.M. | 104.3 |

On post-mortem examination the one that reacted was found to have tuberculosis of both lungs and of the bronchial glands.

All kinds of tales are now going the rounds about tuberculin; it is said to be a dangerous poison, to cause cows in the full flow of milk to diminish or dry off, to cause cows to abort, to cause them to retain the fœtal membranes after calving, and to cause the death of young calves taking the milk from cows being tested.

Of the hundreds of cattle I have tested with tuberculin I have never known this agent to act as a poison; I have known cows that were tested to diminish their milk, to abort, to retain the afterbirth, and young calves to die when the mothers were tested. On the other hand, I have tested cows without there being any diminution in the flow of milk, without their aborting or retaining the afterbirth, and the newly-dropped calves have been as lively as crickets and continued to thrive. Furthermore, we frequently see these direful mishaps and disasters occur among herds that have not been tested with tuberculin; so it seems as though if a herd is tested with tuberculin this agent was blamed for all the troubles that may take place among the stock for at least a year to come, while if the same accidents occurred in a herd that had not been tested no special notice would be taken of them, or other reasons would be assigned.

As to the infallibility of the tuberculin test: Cattle that are very badly diseased sometimes do not react, but generally these cases are plain upon physical examination; then cows advanced in pregnancy may have increased temperatures at times; cows in heat may have a rise in temperature, as may also animals having some sudden febrile disturbance; therefore all these conditions must be borne in mind by the inspector. In other words, the examination of cattle has not yet reached a point where an inexperienced young man can be sent out with a note-book, a supply of tuberculin, a hypodermic syringe and a thermometer to test herds, and then submit his note-book to a cattle commissioner sitting in an arm-chair in his office to say what shall be condemned to death and what shall pass as healthy. The trained expert on cattle and their diseases is still just as necessary and just as valuable a man as he has ever been.

The tuberculin test is of greatest value as an *aid* to diagnosis when used upon the cattle in the quiet surroundings of their homes, as free from disturbing influences as possible. Here the per cent. of error is very slight; in fact, here it is most nearly infallible, nearer infallible than anything we have ever had. The possibility of error is most clearly shown in the following extract from the report of the veterinarian of the Boston Board of Health, just issued this week. These animals were under the excitement of transportation and absence of familiar surroundings.

The first table shows the number of animals condemned as tuberculous since November 20th last, by the Cattle Commission, at Watertown and Brighton, the per cent. of error being 21.1 per cent.

| Animals. | No. killed. | No. tuberculous. | No. not tuberculous. | Percentage not tuberculous. |
|---|---|---|---|---|
| Cows | 96 | 76 | 20 | |
| Bulls | 5 | 4 | 1 | |
| Steers | 3 | 2 | 1 | |
| Total. | 104 | 82 | 22 | 21.1 |

The following table shows the number of animals killed, which failed to react to the tuberculin test, and had been pronounced free from tuberculosis, the Commonwealth brand having been placed upon the right hip:

| Animals | Number killed. | Number tuberculous. | Percentage, tuberculous. |
|---|---|---|---|
| Cows | 62 | 4 | |
| Steers | 1 | | |
| Total | 63 | 4 | 6.34 |

Comment upon these two tables is unnecessary, each speaks for itself, especially the latter one.

Is it a medicine? Another question to be investigated in regard to the action of tuberculosis is, as to whether it has a curative effect; for example, if a herd be tested where tuberculosis exists and a number of cows react; take, for example, a herd of twenty-five native or grade cows, and assume that fifteen react; take those that seem to be badly diseased and kill them, allow five for this; leave those that seem to be slightly diseased (ten) with the ten healthy ones and place them under the best hygienic conditions and disinfect the premises. If in seven or eight months the herd be retested it will be found that five of the ten that reacted before will not react, and that possibly one of the ten healthy ones may react to tuberculin. Or a herd of twenty cows, ten of which were tuberculous eight months ago, now contains but six diseased creatures, the remaining fourteen being healthy, if tuberculin be taken as correct.

Under the law such a herd as I describe would have had fifteen cattle out of twenty-five slaughtered, for which the owner would have received half value, and the carcasses would be thrown away, or under the law as it is hoped it will be, for the farmer's sake, he would receive full value for cows to be thrown away by the State, while it is yet an open question whether ten of them are of no danger, or at least safe to use for beef.

Is it fair, then, to expect the taxpayers to pay full value for the carcasses of what might be termed practically healthy cows, or cows that are safe to use as beef, until this question as to the danger from cows but very slightly diseased, and the curative action of tuberculin upon them, be more fully investigated?

I believe that for the breeder of pure-bred stock the tuberculin test is of the greatest value; it will enable him at once to eradicate all tuberculous animals and breed from only healthy stock; to the breeders of the Channel Island cattle particularly it will prove of inestimable value in leading to the destruction of a lot of weak-constitutioned, valueless animals, and the building up of a stronger, hardier race. For the milkman, however, keeping native or grade cows and constantly buying and selling, I believe that the test alone is too delicate to be of practical value. If I were a milkman and the State wished to test my cows and pay me full value for the cows killed, I should be glad to have it done and to sell stock in this way; but I would wait until the Cattle Commissioners arrived in their official capacity to do the work, and not employ any private practitioners to come and test my herd before the commission was ready to do the work officially. But as a good citizen and a taxpayer I should object to anything that seems wasteful and extravagant until I was sure that it was the wisest course to pursue, especially as we are already an over-taxed people and have enough legitimate uses to put the State's money to without throwing it away. Tuberculosis in cattle is not a disease peculiar to them alone, but it is common to man and many other animals as well. It cannot be "stamped out" like contagious pleuro-pneumonia, but it can be kept down to a minimum by paying more attention to hygiene, cleanliness, sunlight and ventilation, and a gradual weeding-out process in herds where the disease exists. I still believe what I said in a report to the United States Veterinary Medical Association in October, 1893, when I was Chief Inspector of Cattle for the New York State Board of Health: "In suspicious cases when doubt is felt as to whether an animal is tuberculous or not, tuberculin is well worthy a trial; and in herds where a number of cases of tuberculosis are found I believe it is advisable to test the entire herd with it. I do not, however, believe it necessary or practicable to go to every farm in the country and test with tuberculin every cow to be found; but if it be inspected in the ordinary way and found to be healthy, I consider that sufficient.

---

Professor R. S. Huidekoper, by virtue of his connection with the New York College of Veterinary Surgeons, changes his office address to 154 East Fifty-seventh street.

The report of the Board of Cattle Commissioners has just been received. It gives a complete account of the work done in the line of stamping out tuberculosis; a record of all the tuberculin tests with the results obtained; an epitome of the legislation of other States in regard to tuberculosis and their plans of recompense where the animals are destroyed, which makes it a valuable contribution to the busy practitioner as a book of reference. Its strongest point and greatest value lies in the answers of the commission to all leading questions that have arisen under the stamping-out act, and sheds a world of light on the subject to guide other States in dealing with this all-important and intricate subject.

